# Building reproductive health research and audit capacity and activity in the pacific islands (BRRACAP) study: methods, rationale and baseline results

**DOI:** 10.1186/1472-6920-14-121

**Published:** 2014-06-19

**Authors:** Alec J Ekeroma, Tim Kenealy, Boaz Shulruf, Lesley ME McCowan, Andrew Hill

**Affiliations:** 1 South Auckland Clinical Campus, University of Auckland, Middlemore Hospital, Auckland, New Zealand; 2 Office of Medical Education, Faculty of Medicine, University of New South Wales, Sydney, NSW, Australia; 3 Department of Obstetrics and Gynaecology, University of Auckland, Auckland, New Zealand

**Keywords:** Pacific countries, Clinician researchers, Research capacity building, Research skills, Barriers and enablers of research

## Abstract

**Background:**

Clinical research and audit in reproductive health is essential to improve reproductive health outcomes and to address the Millennium Development Goals 4 and 5. Research training, mentoring and a supportive participatory research environment have been shown to increase research activity and capacity in low to middle income countries (LMIC). This paper details the methods, rationale and baseline findings of a research program aimed at increasing clinical research activity and audit in the six Pacific Islands of Fiji, Samoa, Tonga, Vanuatu, Cook Islands and the Solomon Islands.

**Method:**

Twenty-eight clinician participants were selected by the five Ministries of Health and the Fiji National University to undergo a research capacity building program which includes a research workshop and mentoring support to perform research and audit as teams in their country. Data on the participants’ characteristics, knowledge and experiences were collected from structured interviews, questionnaires, focus groups, and an online survey. The interviews and the two focus groups were audio-recorded and all replies were analysed in a thematic framework.

**Results:**

The 28 participants included 9 nurses/midwives, 17 medical doctors of whom 8 were specialists in reproductive health and 2 other health workers. Most (24, 86%) were required to perform research as part of their employment and yet 17 (61%) were not confident in writing a research proposal, 13 (46%) could not use an electronic spreadsheet and the same number had not analysed quantitative data. The limited environmental enablers contributed to poor capacity with only 11 (46%) having access to a library, 10 (42%) receiving management support and 6 (25%) having access to an experienced researcher. Barriers to research that affected more than 70% of the participants were time constraints, poor coordination, no funding and a lack of skills.

**Conclusion:**

Building a research capacity program appropriate for the diversity of Pacific clinicians required research evidence and collaborative effort of key stakeholders in the Pacific Islands and the region. The participants had limited research knowledge, skills and experience and would require individualized training and continuous intensive mentorship to realize their potential as clinician researchers for their services in the Pacific.

## Background

Health research and clinical audit are fundamental components of functioning health systems as they are important at addressing Millenium Development Goals (MDG) and quality of health service delivery [1-4]. Clinical research and audit are implicit in all health service functions and contributes to the effectiveness and efficiency of health care [5]. Low to middle income countries (LMIC) have a disproportionate lack of resources, capacity and personnel leading to poor research output and utilisation [6,7].

Although there has been agreement by Ministers of Health, accompanied by policy efforts of international agencies such as the World Health Organization, (WHO) to strengthen research system capacity in LMICs [2,8,9] there is evidence that political commitment has not matched the rhetoric as poor research capacity in LMIC persist [10].

There have been rewarding partnerships between funding agencies, research institutions and individuals in high income and LMIC resulting in the generation of successful collaborative models [11-13]. Successful research capacity building (RCB) programs tend to be located at dedicated research institutions [14] and universities although systems and sustainability are often weak where there is no reciprocal local funding [15]. Such RCB programs are commonly aimed at developing research scientists [16] with only a few targeting clinicians [17]. While many agencies have concentrated efforts at developing research systems capacity from top down, an effort should also be made to develop research capacity from the bottom up [18]. Such an approach acknowledges the clinicians role in identifying research priorities at the clinical interface [19,20].

Training clinicians to perform clinical research, in the absence of research scientists, requires a paradigm shift [21,22]. Clinicians, who are mostly nurses and doctors, have an advantage as clinical researchers over non-clinical researchers as they are likely to have better understanding of the research questions, are able to collect demographic and patient data, and are more enthusiastic in applying locally derived evidence to patient care [23,24]. There is the view that clinicians performing research make better clinicians [25].

There have been only four quasi- experimental studies with different interventions targeting clinicians in LMIC. Lessons from a funded RCB program of rural general practitioners in Australia failed to demonstrate expected outputs, probably due to the lack of time allocated to research and administration support [26]. Similarly, a RCB for primary care in the United States failed, with evaluators recommending protected time for research and sustained mentoring [27]. On the other hand, in-service training at teaching hospitals in Vietnam was deemed effective and sustainable [20] and an allied health RCB program in Australia was found to be effective in generating research publications [28]. It seems that successful RCB programs include substantial research training, completion of a project supported by mentors [29] and strong scientific leadership [30].

To better understand the nature of individual interventions within a RCB program and the characteristics of the clinician participants required to increase clinical research and audit activity and capacity in LMIC, prospective studies are needed where the literature, professional peers and stakeholders’ perspectives guide the shape of RCB programs [31].

### A RCB program in reproductive health in the pacific

As with most LMIC, the 23 small LMIC in the Pacific Ocean have weak health research systems, limited human, infrastructure and financial resources in all disciplines compounded by geographical isolation and burgeoning population growth [14,32-35]. Many of these countries are not on track to achieve MDG 4 (reducing child mortality by two-thirds) and 5 (reducing maternal mortality by three-quarters) [36]. Three women die in the Pacific every day due a pregnancy related problem [37].

Pacific leaders in reproductive health decided at the 2007 meeting of the Pacific Society for Reproductive Health (PSRH) that member clinicians in the discipline are to be encouraged to perform clinical research and audit to improve local evidence in policy, service provision and as a way to address MDG 4 and 5. Research and audit workshops for clinicians commenced in 2009. The PSRH is a Charitable Trust registered in New Zealand with a membership of doctors, midwives, nurses and allied health professionals working in thirteen developing countries of the Pacific. The Building Reproductive health Research and Audit Capacity and Activity in the Pacific islands (BRRACAP) Study will assist the PSRH and policy makers in the Pacific region understand the impact of a RCB program aimed at clinicians and at improving reproductive health outcomes.

### Selection of countries

Five Pacific Island countries - Vanuatu, Solomon Islands, Fiji, Samoa and Tonga - were selected and invited purposefully, and a sixth, Cook Islands, was included following a request from their health service. The five independent nations were chosen to represent the diversity of cultures, challenges and systems: Melanesian (Vanuatu, Fiji, Solomon Islands) and Polynesian (Samoa, Tonga) countries; populations that were more than 500,000 (Fiji, Solomon Islands) and smaller populations (Samoa, Vanuatu, Tonga); without a university (Tonga, Solomon Islands); with a medical school (Fiji, Samoa). The Micronesian group of Islands that are further north in the Pacific were not invited due to the anticipated high cost of participation. The Ministries of Health of five countries agreed to participate as a health service and a stakeholder. Discussions with the Fiji National University (FNU) led to their engagement replacing the need to engage the Fiji public health service.

### Research workshop

The workshop incorporated 48 hours of seminars, lectures and small group work over 6 days. The content was devised with the aim to teach the basic components of clinical research and audit and included motivational talks aimed to inspire the participants to believe that doing research was possible even for those who had not done this previously. The participants from the same country were encouraged to work together as a team to develop projects they identified as priorities to their service. The program with listed faculty is attached (Additional file 1). The workshop was conducted in Auckland New Zealand to utilise the wealth of experienced Pacific researchers from various academic institutions especially from those at the University of Auckland.

### Research mentoring

The participants were asked to nominate a preferred research mentor from a list of mentors, which included some of the faculty of the research workshop. All the sixteen research mentors who consented to participate are established researchers and all except five have Pacific research experience. The five mentors without Pacific experience were encouraged to liaise closely with the PI who became the co-mentor to their participants.

A Code of Mentorship for mentors and participants was adapted from Blixen [38] to guide the mentor-mentee relationship. It was not meant to be prescriptive and an informal mentoring relationship works better for adult learners [39]. A participatory action learning philosophy was encouraged where the mentor and participant learn from each other during the research journey. This was done by frequent email reminders to both mentors and participants to exchange ideas and thoughts about research progress, assistance, barriers, enablers and ideas.

The quality of mentoring and the performance of the participants will be evaluated at the conclusion of the formal part of program in August 2014, 18 months after inception.

The main aim therefore of the BRRACAP Study was to determine the impact of a RCB program on research activity amongst selected reproductive health clinicians in the participating countries. The secondary aim was to understand the characteristics of those clinicians who become research successful and the barriers to and enablers of clinical research in the Pacific Islands.

## Methods

### Study participants

The five Ministries of Health were invited to select five reproductive health clinician participants for the study. Due to funding constraints, the Cook Islands health service was asked to provide only one participant. We assumed that five clinicians, made up of midwives, doctors and others, working on a clinical research or clinical audit project together would provide team support, different skills and enough members for task delegation, i.e. critical mass. Critical mass is the capacity required to execute collective research action and is dependent not only on numbers but the commitment, costs, and skill set of the individuals in the group. We planned that 26 participants from the six health services would attend the research workshop; which would be manageable in both class size and cost, and would provide an adequate sample to follow for two years even with minor attrition.

The requirements for the participants were: 1) an informed consent to participate; 2) active clinician (doctors, midwives, nurses and clinician managers) working in reproductive health; 3) must want to learn and do research/clinical audit; and 4) preferably, be in a leadership role, a team player, have performed research, and a member of the Pacific Society for Reproductive Health (PSRH). All midwives in the Pacific were initially trained as nurses.

To inform the content of the workshop, a needs analysis survey of selected personnel was conducted using SurveyMonkey® [40]. The invited personnel were Pacific stakeholders, reproductive health clinicians, research funding agencies in the region and selected researchers who had done research in the Pacific.

### Stakeholders

The key stakeholders were the Ministries of Health in the five countries namely Vanuatu, Solomon Islands, Samoa, Tonga and the Cook Islands and the FNU. The other two key stakeholders were the PSRH and the Faculty of Medicine and Health Sciences of the University of Auckland. The former provided the mechanism and the umbrella under which the study could be performed and the latter as the provider of main faculty for the research workshop and the supervision and advice for the study.

### Employer’s commitment

The employers, through the managers of the services, agreed to support the participants with their research projects by funding airfares and stipends to the research workshop, allocating half a day a week for the participant to perform clinical research and audit activity and providing their participants with internet access for research.

Ethical approval was gained for this study from the University of Auckland Human Participants Ethics Committee (Ref. No. 8373).

### Data collection

The RCB program was designed to follow a flow of interventions, tasks and objectives as in Additional file 2.

### Mapping of research systems

Senior representatives of the six countries were invited to complete the Mapping of Research Systems using the tool that was used by WHO and COHRED for mapping research systems in 15 Pacific countries in 2007 [41]. Five were completed prior to the research workshop in March 2013. The mapping aimed to assess the quality of each country’s health research systems and how that compared to the 2007 review. It also served to understand the research culture in each country and how that would relate to the performance of their participants.

### Characteristics questionnaire

All the participants were asked to complete a ‘Characteristics Questionnaire’ by the first day of the research workshop (Additional file 3). The questionnaire was developed using a slightly modified tool that was validated in in Australia [42] and made it applicable for the Pacific clinicians. The tool was tested using cognitive interviewing [43] of five clinicians. The questionnaire sought demographic professional and personal data that will inform of the participants’ research experience, their current qualifications, work role and research expectations, and what they perceived to be barriers to and enablers of research in their setting. The questionnaire was piloted and could be completed comfortably within 30 minutes with responses collated thematically.

### Interviewer-administered questionnaire

All the participants were interviewed by one of the three trained health-worker interviewers. The structured interviews were conducted face to face on the day before the workshop and lasted about 15 minutes each. The interview was semi-formal which the interviewer could elaborate if needed. It sought to understand the participant’s current professional commitments, their organisational research culture, research experience, future research plans, and personal information that impacts on research effectiveness. A research skills test was performed that required some knowledge in using a Microsoft Excel spread sheet. The interview data also cross-checked the information gathered in the mapping and professional questionnaires. The questionnaire was piloted using cognitive interviewing methodology, interviews were audio-recorded, independently transcribed, content checked against original recording, offered to participants to review, and then analyzed using key response patterns and themes. Mixtures of deductive and inductive approaches were used to identify themes consistent with the question schedule embedded in the data. The data was coded and emerging themes were used to build a thematic framework, to which the participant responses were categorized [44].

### Focus groups

Heads of departments, clinical managers and specialists were invited to two focus groups of which one was for the midwives (six midwives and one nurse) and one for the doctors (eight doctors). The focus group interviews were facilitated by one researcher (AE) over two evenings lasting 2 hours each and sought to obtain a consensus on how research performance should be measured in the two groups. The focus groups were designed to identify research outcomes that were important to the clinicians. These were audio-recorded, independently transcribed into verbatim script, offered to participants to review, then analyzed using key response patterns and themes [44].

A set of research indicators, or surrogate markers of research performance was developed by the groups and was then presented to the whole group for feedback. Further development was done before these were sent to all the stakeholders for comment and as part of continuing consultation. Maximum points were assigned by consensus to identified research outputs that were of most value to the participants and decreasing points to those research activities which were deemed of less value. The concept of points for an assigned professional activity was made easier by an elaboration on a continuous professional development points system most of the participants were familiar with.

### Pre-test and post-test

The Pre and Post Test Questionnaires were similar and were designed to assess knowledge of research methodology and principles prior to and after the workshop. It also sought to explore their awareness of routinely collected data in their settings and how these can be analysed for information that can guide service delivery. The Post-Test Questionnaire had additional questions, which assessed attitude to research and was obtained from the work of Bates in Ghana [45]. This was in the form of short answers in an open text format and was piloted using cognitive questioning.

### Evaluation and feedback

As well as daily reflections on the learning from the workshop topics obtained by interactive discussion, participants completed an evaluation questionnaire on the last day of the workshop.

### Group mentoring and social media

Group mentoring is important for group learning so that the same advice to one participant or one question can be shared for the others learning [46]. The wisdom and experience of others is shared with the group [47]. Opportunities were created for group mentoring using social media in Facebook, Research Gate and Linked In. These platforms for group mentoring are in place although they have not been evaluated [47]. All participants and mentors were invited to join one or all three sites. Mentoring by email, Skype and by phone was also encouraged although email was considered cheaper. The study design and outcome measures are subject to change during the study as required by the participatory action learning process [48].

## Results

This paper reports on the baseline results, which were captured from the Characteristics Questionnaire.

### Participants

The 28 clinicians selected from the six participating Pacific Island countries: Cook Islands (1), Fiji (8), Samoa (4), Solomon Islands (5), Tonga (5) and Vanuatu (5). A participant chosen by Tonga and who was studying in NZ had approved leave for only 2 days of the workshop.

Most of the participants were medical practitioners (17, 61%) although most had various roles. Most had a postgraduate diploma or a Masters degree (23, 82%) (Table 1). The age range of participants was 28 to 55 years of age with a median age of 40. There were 20 women and 8 men.

**Table 1:** Research workshop participants by profession, qualification and role descriptions (N = 28)

**Characteristics of participants**	**N**	**%**
**Profession**		
Midwife	8	29
Nurse	1	4
Obstetrician gynaecologist	8	29
Medical doctor	9	32
Other	2	7
**Qualification**		
Basic/minimum registrable qualification	5	18
Postgraduate diploma	12	43
Postgraduate masters	11	39
**Roles (each participant may have more than one)**		
Academic	6	21
Patient care	24	86
Teaching	20	71
Supervision	24	86
Administration	18	64
Research as part of role	24	86

### Confidence, experience and access

Two (7%) of the participants were ‘not confident’ to use a computer, 10 (36%) were ‘not confident’ or ‘less confident’ to find a paper at the workplace and that 17 (61%) were not confident to write a research protocol (Table 2). Of the 11 participants with Masters degrees, 5 (45%) were ‘not confident’ to use a referencing software and the same proportion were ‘not confident’ to write a research proposal. Although 8 (29%) had submitted a proposal to an Ethics Committee, only 2 (7%) had written a publications in peer-reviewed journals when they were working in Australia and NZ (Table 3). Only 13 (43%) of the participants had skills to use an Excel spread sheet (Table 4), which included 5 (45%) of the 11 participants with Masters degrees. Of the 24 who were expected to perform research as part of their role, 11 (46%) had access to a library, 10 (42%) felt management was supportive and 9 had access to administrative assistance (Table 5).

**Table 2:** Level of confidence in various tasks and skills (N = 28)

**Skills scale**	**Not confident (0–2)**	**Less confident (3–4)**	**Confident (5–6)**	**Very confident (7–8)**	**Extremely confident (9–10)**
Using a computer referencing system	11 (39%)	5 (18%)	6 (21%)	3 (11%)	3 (11%)
Writing a research protocol	10 (36)	7 (25)	2 (7)	9 (32)	0
Providing advice to less experienced researchers	9 (32)	6 (21)	5 (18)	6 (21)	2 (7)
Reading a paper critically	8 (29)	4 (14)	7 (25)	6 (21)	3 (11)
Finding a paper at workplace	7 (25)	3 (11)	6 (21)	7 (25)	5 (18)
Using a word processing software	2 (7)	1 (4)	6 (21)	4 (14)	15 (54)
Using a computer to write a letter	2 (7)	0	3 (11)	3 (11)	20 (71)

**Table 3:** Research skills (N = 28)

	**N**	**%**
Obtained research funding	2	7
Written a publication in a peer-reviewed journal	2	7
Had clinical audit training	5	18
Analysed qualitative data	7	25
Submitted an ethics application	8	29
Written a letter or an article in any local publication	10	36
Had research training	13	46
Analysed quantitative data	13	46
Written a research report	14	50
Integrated research findings into every day practice	14	50
Designed a questionnaire	19	68
Used a computer data management system	24	86
Collected data	26	93

**Table 4:** Observed level of skill using a MS Excel spreadsheet

**Skills assessment**	**N = 28**	**%**
Extremely skilled	1	0
Very skilled	9	32
Skilled	3	11
Not skilled	3	11
Extremely not skilled	12	43

**Table 5:** Research part of job and have access to (N = 24)

	**N**	**%**
Library	11	46
Management support	10	42
Administrative assistance	9	36
Software	7	29
Experienced researcher	6	25
Equipment	6	25
Regular research/audit meetings	6	25
Research office	5	21
Supervision	4	17
Funding	4	17

Nineteen (68%) had not published or presented a paper at a conference. Only two had presented more than once and only one had published more than one paper. Eighteen (64%) were not performing any current research and three (11%) had more than one current project.

All of the participants agreed or strongly agreed that research was needed and in their department and 27 felt the same for clinical audit. Eighteen (64%) agreed or strongly agreed that research evidence was used to inform practice at their work.

All wanted to learn how to do audit however two participants did not want to learn to do research.

### Barriers and motivators for research

Nearly all the participants identified time constraints as a barrier to performing research although the lack of research coordination, funding and skills also featured highly (Table 6).

**Table 6:** Barriers and motivators to performing research (n = 28)

**Barriers**	**Yes**	**%**	**Motivators**	**Yes**	**%**
Time constraints	27	96	Develop skills	25	89
No coordination	22	79	Mentors available	24	86
No funding	20	71	Problem identified and needs changing	22	79
Lack of skills	20	71	Keep brain stimulated	21	75
Lack of administration support	18	64	Increase satisfaction	20	71
Lack of software	18	64	Link to university	20	71
Lack of backfill	16	57	Career advancement	19	68
Fear of research language	14	50	Opportunity to participate at own level	19	68
Lack of support from management	14	50	Colleagues doing research	18	64
Prefer work life balance	14	50	Grant funding	18	64
Equipment	14	50	Formal part of postgraduate study	17	61
Other personal commitment	12	43	Encouraged by managers	16	57
Fear of getting it wrong	9	32	Desire to prove a theory	16	57
Isolation	8	29	Dedicated time to research	15	54
Not interested in research	7	25	Study or research scholarship	15	54
			Research written into role description	13	46
			Other	13	46

Motivators for doing research were to develop skills, mentor availability and solving problems.

## Discussion

### The BRRACAP program

Only a few RCB programs have targeted clinicians and our study is the first prospective study to provide a RCB program, which includes research training and mentorship to a large number of clinicians from LMIC in the Pacific. It was important to collaborate with key stakeholders, as there were resource implications and responsibilities for staff support on their return home from training.

Our study was well supported by the health services of the five Pacific LMIC, the Dean of FNU and funders. Development partners and governments understand the importance of developing research capacity in LMIC as a pre-requisite to improving health outcomes [13,49-52] and addressing the gaps in MDG 4 and 5 [53]. However, most of the 19 recommendations from a WHO sponsored RCB workshop in the Pacific in 2007 [35] have not been implemented with most countries in the region continuing to have workforce challenges [54] and weak health research systems [14].

Pacific values of reciprocity, collectivity, respect, generosity, importance of relationships as they relate to the various Pacific Research Methodologies was emphasised at the workshop. This was seen central to strengthening capacity across very diverse social, cultural, language, historical, axiological, ontological and epistemological standpoints of Pacific Islanders. Some of the Pacific research frameworks such as the *Talanoa*[55] and *Faafaletui*[56] frameworks, encapsulates Pacific values and traditions using cross-cultural storytelling and perspectives derived from Pacific traditions and values. The participants were encouraged to consider these frameworks in their research work and to be aware of the “importance of carrying out research alongside their day-to-day duties” [33].

The framework of the RCB program was guided by research evidence that training of clinicians as researchers require didactic training in core topics and clinical research methods [20,57,58] supported by an enhancing environment of collaborations [31,59] and appropriate mentorship [26,27,38]. We endeavoured to provide these essential elements of RCB within the constraints of time, funding, distant mentoring and the barriers at the participants’ home environment.

### The results

The 28 reproductive health clinicians, selected by their managers or employers, came from varying educational and professional backgrounds. It was important in this first study, to observe the impact of a RCB program on a range of clinicians including nurses. Whereas the few RCB programs have targeted medical doctors, who were well represented in our study (17, 61%), there is a need to develop nurse-led research in LMIC [60]. Nine (33%) nurses were selected of which three were from the FNU and the others were leading midwives in their services.

Selection criteria were agreed with the employers and on face value, the 28 participants were highly regarded reproductive health clinicians as reflected by the fact that 23 of them had a postgraduate Diploma or Masters qualification. However, it was concerning that 3 (11%) were not confident in using a word processing software, 15 (44%) did not know how to use an Excel spreadsheet and two nurses did not want to learn how to do research. The selection of staff for overseas workshops from the Pacific Islands can be challenging as there is a skills shortage, therefore the selection takes other factors into account and tend to favour those in senior management, who view a funded trip as a work reward (Wame Baravilala, personal communication). Although there are no clear criteria for selection of clinicians for research training, the WHO Training in Tropical Diseases Research Program have selected “young and talented scientists” who submit acceptable research proposals [30]. Attaining higher research training however does not guarantee satisfactory research output [61].

A third of the participants were nurses and it is essential to monitor the impact of educational interventions on this important workforce despite the observation that more research publications were authored by doctors rather than by nurses [14]. Important factors that limit nurse participation in research are a lack of access to research training and infrastructure compared to doctors including hierarchies of power among disciplines [60]. An increase in research by nurses would improve the quality of nursing care through an increase in evidence utilization [62]. Educational needs, motivators and barriers for research may be different for nurses.

Although 26 had collected data (Table 3) only 13 (46%) can use basic functions of an Excel spreadsheet and the same number have analysed qualitative data. Twelve (43%) were not confident to read research articles critically and 17 (61%) were not confident in writing a research proposal. Despite 24 (86%) clinicians being required to perform research as part of their employment, only 11 (46%) had access to a library and 6 (25%) to an experienced researcher. Conversely, with limited research resource, more barriers and fewer enablers in the Islands, publication output is stifled despite 6 (25%) of those expected to perform research recording access to an experienced researcher. Of the 6, 3 were nurses and the other three were junior medical staff and they often view their consultant specialists as experienced researchers. Seven of the eight specialists had not published or lead a research program. This confirms previous findings that research in the Pacific is hampered by not only a lack of research infrastructure but by the lack of clinicians with research skills and knowledge that is required to perform research [14,33,35]. It also showed a weakness in the specialist training curriculums in the Pacific.

The participants other roles expected of them as leaders of their departments and teams pose time constraints on research activity with 27 (96%) (Table 6) identifying time constraints as a major barrier as other RCB studies have identified [63,64]. We requested of the participants’ employers that half a day a week per allocated for research and audit activity. There is evidence however that clinicians may fill this time with clinical work as they are more comfortable in doing that than research [26]. Other significant barriers were the lack of skills (20, 71%) and lack of administration support (18, 64%), which was the reason why it was requested that the employers provide support to their researchers. Addressing the barriers may require modest resources but will be the price for a research-aware, if not a research-skilled workforce. Surrogate markers of research developed by the group will assist in assessing the participants progress with their research objectives.

The commonest motivating factors for the participants were the development of research skills (25, 89%) and the availability of mentors (24, 86%). Research skills and knowledge have traditionally been delivered to clinicians as postgraduate courses such as a Masters degree or in a workshop format such as the one designed for this study [17,45,65]. Other modes of delivery such as video linking [66] and in-service training were found effective [67] but were deemed not suitable or possible for this study. The mentoring program was designed to be responsive to the participants needs. Most of the participants would need significant assistance with their identified research or audit projects so the experienced research mentors of their choice was considered preferable. Most of the mentoring will be by email and online and this has been shown to be effective in other settings [68]. The creation of mentoring on social media to provide group learning may provide another opportunity for research support with the knowledge that those with limited internet access may not be served by this medium.

Strengths of our study include the broad range of clinicians selected, the enthusiasm of the Ministries of Health to participate and the participants to learn. The limitation of the study was the uncertainty in selection criteria that resulted in the selection of two participants who did not want to learn research and the severe limitation in knowledge and skills of some, which may hamper their learning of basic research principles. In addition, the participants needed individualised and local intensive mentoring so a participatory action learning (PAL) and supportive local environment can be provided. The PAL methodology would have been suitable for this study [69] and has been used successfully in two areas of the Pacific [70,71] although long term research outputs and outcomes were not measured.

## Conclusion

Fewer than five studies have published primary data describing RCB programs for clinicians in LMIC and a need was identified to improve reproductive health outcomes in the Pacific Islands. Training and supporting frontline clinicians to perform health research and audit and organising a RCB program across the diversity of small Pacific Island states with different cultures, capacities, priorities, resources and systems was challenging. The success in launching this RCB program depended on the collaboration and support of key stakeholders in the six Pacific states and the region.

The selected clinicians were a mixed group and besides the workshop training given, all will require individualized intensive mentoring and other support, tailored for research or audit projects that are closer to their area of clinical knowledge and expertise. It will be unrealistic to expect all the participants to perform major research tasks but if a number of important barriers and enablers of research, which were identified were addressed and cultivated respectively, then the participants may, with adequate mentorship, increase research and audit activity and build research capacity in the six Pacific Islands.

This paper describes the methods, rationale and baseline findings for the BRRACAP Study. Through detailed exploration of personal, professional and environmental factors that impact on research performance, our findings suggest that this RCB program has the potential to lead to a better understanding of personal and professional factors that may inform RCB programs in the future. Further findings from this RCB program will be reported in future publications.

## Competing interests

The authors declare that they have no competing interests.

## Authors’ contributions

AE participated in the conception, design and coordination of the study, performed the literature search, data collection, analysis and drafted the manuscript. TK participated in the design of the study, performed the literature search, conducted review and helped to edit the manuscript. BS participated in the conception of the study, assisted with study design and helped edit the manuscript. LM participated in the design of the study and helped to edit the manuscript. AH participated in the conception of the study, assisted with study design and helped edit the manuscript. All authors read and approved the final manuscript.

## Pre-publication history

The pre-publication history for this paper can be accessed here:

http://www.biomedcentral.com/1472-6920/14/121/prepub

## Supplementary Material

Additional file 1 Research Workshop Program. Click here for file

Additional file 2 Interventions, Tasks and Objective of the BRRACAP Study. Click here for file

Additional file 3 Professional and Personal Characteristics’ questionnaire. Click here for file
