# Supplementary material for: Building reproductive health research and audit capacity and activity in the pacific islands (BRRACAP) study: methods, rationale and baseline results
Source: BMC Med Educ. 2014 Jun 19;14:121. doi: 10.1186/1472-6920-14-121 (PMC4069343; doi:10.1186/1472-6920-14-121)
Supplement: Additional file 1 — Research Workshop Program. [file 1472-6920-14-121-S1.docx]

Appendix 1: Research Workshop Program

**Day 1: General Concepts in Research** (with Pacific contextualization)

| 0800-0900 | What is research? Why is it important? Why priorities?  *Dr Teuila Percival, Pacific Health, University of Auckland (UoA)* |
| --- | --- |
| 0900-1000 | What are the essential skills and tools for research?  *Ms Sharon Biribo, Health Research Support, FNU* |
| 1000-1030 | Morning Tea |
| 1030-1130 | From Ideas to a Research Plan  *A/Prof Boaz Shulruf, University of New South Wales (UNSW)* |
| 1130-1230 | Essential Epidemiology  *Prof Robert Scragg, Population Health, UoA* |
| 1230-1330 | Lunch |
| 1330-1430 | Epidemiology and Quantitative Research  *Prof Robert Scragg, Population Health, UoA* |
| 1430-1530 | Support needed for Pacific research and by researchers.  *Dr Nuhisifa Seve-Williams & Dr Etuate Saafi, Pacific Health Research Council* |
| 1530-1630 | Participants break into smaller groups to discuss research in the Pacific, barriers and support needed  *Alec, Aumea, Teuila, Vili, Rajat, Ofa, Sharon, Peggy, others* |
| 1630-1730 | Reflections on the day |
| 1900-2000 | Optional sessions - small groups - need to book  How to read a Journal  *Alec Ekeroma*  How to do a literature search  *Megan Clark* |

**Day 2: Data Management**

| 0800-0900 | What data are you collecting in the Pacific?  How to collect and use data for research?  *Prof Rajat Gyaneshwar, School of Medicine, Fiji National University* |
| --- | --- |
| 0900-1000 | How to gather and organise data for research?  *Dr Ofa Dewes, Pacific Health, UoA* |
| 1000-1030 | Morning Tea |
| 1030-1130 | Introduction to statistics in medicine 1  *Dr Arier Lee, UoA* |
| 1130-1230 | What is Qualitative Health research?  *Dr Vili Nosa, Pacific Health, UoA* |
| 1230-1330 | Lunch |
| 1330-1430 | Introduction to statistics in medicine 2  *Dr Arier Lee, UoA* |
| 1430-1530 | Pacific research methodologies and People participating in research  *Dr Malakai Ofanoa, Pacific Health, UoA* |
| 1530-1630 | Participants break into country groups to discuss data and skills needed for various research designs  *Alec, Aumea, Teuila, Vili, Rajat, Ofa, Sharon, others* |
| 1630-1700 | Country presentations – 10 minutes each |
| 1800-2100 | Focus Group Meeting - selected midwives/nurses |

**Day 3: Research Design**

| 0800-0900 | Which Research Method?  *Dr Judith McCool, UoA* |
| --- | --- |
| 0900-1000 | Questionnaire Design  *A/Prof Tim Kenealy* |
| 1000-1030 | Morning tea |
| 1030-1130 | Surveys  *Prof Peggy Fairbairn-Dunlop* |
| 1130-1230 | Measurement Tools  *A/Prof Boaz Shulruf, UNSW* |
| 1230-1330 | Lunch |
| 1330-1430 | The Place of Ethics in Research & what we look for in a research proposal  *Ms Sharon Biribo, Research Manager, FNU* |
| 1430-1530 | Narrative Methods in Health Research.  *Prof Peggy Fairbairn-Dunlop, Chair, Pacific HRC* |
| 1530- 1630 | Participants break into country groups to discuss research topics, research designs and ethical issues in the Pacific  *Alec, Aumea, Peggy, Teuila, Vili, Rajat, Sharon, others* |
| 1630-1700 | Country presentations: different presenters |
| 1800 | Focus Group Meeting - selected doctors |
| 2000-2200 | Optional session - small groups - please book  How to read a Journal  How to do a literature search |

**Day 4: Clinical Audit & Research Leadership**

| 0830-0930 | Importance of Clinical Audit in Quality Improvement.  *Mr Alan Wilson, CEO Alliance Health+* |
| --- | --- |
| 0930-1030 | How to perform an audit project and the audit cycle  *Dr Alec Ekeroma* |
| 1030-1100 | Morning tea |
| 1100-1200 | Research Leadership and Team Building  *Prof Andrew Hill, SACS, UoA* |
| 1200-1230 | How to write a Research Proposal  *A/Prof Tim Kenealy* |
| 1330-1430 | How to Develop Clinical Practice Guidelines 1  *Prof Cindy Farquhar, Cochrane Collaboration, UoA* |
| 1430-1530 | How to Develop Clinical Practice Guidelines 2  *Prof Cindy Farquhar/Prof R Gyaneswar* |
| 1530-1630 | Participants break into smaller groups to discuss audit projects and what is doable in the Pacific  *Alec, Rajat, Sharon, Tim, PhD students* |
| 1630-1730 | Reflection on research leadership  *Alec, Rajat, Sharon* |

**Day 5: Research Synthesis, Guidelines, Dissemination**

| 0800-0900 | Research Projects and Experiences with Pacific people in South Auckland  *Dr Debbie Ryan*, Pacific Perspectives; *A/Prof Tim Kenealy, UoA* |
| --- | --- |
| 0900-1000 | Barriers and Enablers in Research  *Dr Alec Ekeroma, UoA* |
| 1000-1030 | Morning tea |
| 1030-1130 | How to perform an audit project and the audit cycle  *Dr Alec Ekeroma* |
| 1130-1230 | How to implement clinical audit findings  *Prof Rajat Gynaeshwar* |
| 1230-1330 | Lunch |
| 1330-1430 | Does your research/audit project need funding?  *Ms Sharon Biribo/ Dr Alec Ekeroma* |
| 1430-1530 | What Research Skills are Needed and How to Acquire them  *Dr Teuila Percival*/*Ms Sharon Biribo/Rajat Gyaneshwar* |
| 1530-1630 | Participants break into country groups to discuss Guidelines Development  *Alec, Rajat, Teuila, Vili, others* |
| 1630-1700 | Country presentations: Guidelines Needed |
| 1700- 1800 | Tour of Middlemore Hospital |

**Day 6: Reflection & Recapture**

| 0800-0900 | Research Findings into Action  *Dr Teuila Percival* |
| --- | --- |
| 0900-1000 | Research Networks and Collaborations  *Prof Lesley McCowan, Head, Department of O&G, UoA* |
| 1000-1030 | Morning tea |
| 1030-1130 | How to Write Research Reports and Publications 1  *Prof Shanthi Ameratunga, PopHealth, UoA* |
| 1130-1230 | How to Write Research Reports and Publications 2  *Prof Shanthi Ameratunga, PopHealth, UoA* |
| 1230- 1330 | Lunch |
| 1330 - 1430 | Questionnaire  Complete interviews  Discuss project work |

**Day 7: Projects & Outcomes**

| 1000-1100 | Essentials - research tools, communication, project management  *Dr Aumea Herman* |
| --- | --- |
| 1100-1230 | Country research projects  Vanuatu, Solomons, Tonga, Samoa, Fiji  *Drs Ekeroma, Rajat, Nosa, Percival, Ofanoa, Sharon, Dewes* |
| 1230-1330 | Lunch |
| 1330-1430 | Individual projects  Participant presentations – discussing methodology  *All* |
| 1430-1600 | Expectations of the Project  Monitoring & Evaluation  Mentors and Support  Post workshop test  *Dr Alec Ekeroma* |
| 1600 | Farewell |
