# Supplementary material for: Building reproductive health research and audit capacity and activity in the pacific islands (BRRACAP) study: methods, rationale and baseline results
Source: BMC Med Educ. 2014 Jun 19;14:121. doi: 10.1186/1472-6920-14-121 (PMC4069343; doi:10.1186/1472-6920-14-121)
Supplement: Additional file 3 — Professional and Personal Characteristics’ questionnaire. [file 1472-6920-14-121-S3.docx]

**Appendix 3: Professional and Personal Characteristics’ questionnaire**

**A. Research Experience**

We need to ask you some questions about your research or audit experience. For the following several questions, please choose a number from 0 to 10 and write it next to the statement to indicate how much you agree with the statement.

| 0 | 1 | | 2 | | 3 | | 4 | | 5 | 6 | 7 | | 8 | | 9 | | 10 |  |
| --- | --- | --- | --- | --- | --- | --- | --- | --- | --- | --- | --- | --- | --- | --- | --- | --- | --- | --- |
| No Skill | |  | |  | |  | |  | |  | |  | |  | | Extremely Skilled | | |

1. ____Do you know how or where to find a research paper to refer to at your workplace?
2. _____Do you know how to read a research paper critically?
3. _____Do you use a computer referencing system? (e.g. Endnote)
4. _____Do you know how to write a research protocol?
5. _____Have you secured research funding before?
6. _____Have you submitted an ethics application before?
7. _____Have you designed questionnaires?
8. _____Have you collected data before? e.g. audit, surveys, interviews
9. _____Have you used a computer data management system? eg. Excel
10. _____Have you analysed qualitative research data?
11. _____Have you analysed quantitative research data?
12. _____Have you written a research report?
13. _____Have you written a letter or an article in any local publication? eg hospital newsletter, newspapers, magazines, etc
14. _____Have you written a publication in peer-reviewed journals?
15. _____Have you integrated research findings or findings form a journal into your every day clinical practice?
16. _____Have you provided advice to less experienced researchers?
17. _____Have you had research training?
18. _____Have you had clinical audit training?
19. _____Have you performed an audit project?

**B. Current/recent research**

1. Please list all the names of research published/presented in the last 12 months. If more than 5, please list at back of form.

| Name of Paper | Your role in project | Where published/presented |
| --- | --- | --- |
|  |  |  |
|  |  |  |

2. Please list all the names of audit projects published/presented in the last 12 months. If more than 5, please list at back of form.

| Name of Paper | Your role in project | Where published/presented |
| --- | --- | --- |
|  |  |  |
|  |  |  |

3. Please describe any research/audit projects you are currently doing?

**C. Individual Participants Details:**

This information is important to assist in designing a programme for support for you to complete agreed research/audit skills/projects.

Full Name:_______________________________________

Date of Birth: ___/___/___ Gender: Male/Female

Relationship status: Married/Single/Partner/Other

Number of dependents:__________

Institution/Hospital: _____________________________

How many beds approximately in your hospital?________

Town: ________________________Country: ______________________

Current job/position/role: _____________________________

Detail of your work description/roles:

(examples: supervise, teach, care for patients, etc)

1. ___________________
2. ___________________
3. ___________________
4. ___________________

How long have you been in your current role? _______

Your qualifications:

Qualification/Certificate Year Institution

| 1. |  |  |
| --- | --- | --- |
| 2. |  |  |
| 3. |  |  |

**D. Research Activities as Part of Your Role**

Is performing research/clinical audit part of your role? _Yes/No_____

How many hours in a 40 hour week are you permitted to do research/audit?________

If your answer is Yes, then please answer the questions below with a tick if you agree with the statement. If you are not expected to perform research, then skip this Section.

☐ Access to a computer and email most of the time

☐ Access to a researcher

☐ Administration support

☐ Library access

☐ Management support

☐ Regular meetings to discuss research activities

☐ Research funding

☐ Research officer

☐ Research supervision

☐ Software

☐ Research equipment

Other, please specify/Or any other comments:

**E. Personal Barriers and solutions:**

Do you think your hospital/institution need to do research? Yes ____ No ____

Do you think your hospital/institution need more audit? Yes ____ No ____

Do you think research and audit are used to

inform health care in your hospital? Yes_____No_____

It Yes, give three examples of research being used in your work area (eg name of guidelines, protocols)

1. _________________________________________
2. __________________________________________
3. __________________________________________

Do you want to learn how to perform clinical audit? Yes____ No____

If Yes, why do you want to do audit?

1. ___________________________________________________
2. ___________________________________________________
3. __________________________________________________
4. ___________________________________________________

Do you want to learn how to perform research? Yes____ No____

If Yes, why do you want to do research?

1. _____________________________________________________
2. _____________________________________________________
3. _____________________________________________________
4. _____________________________________________________

Do you think there are barriers in you performing research/audit where you work?

 Yes ____ No ____

If your answer is Yes, then what are those barriers?

1. ___________________________________________________
2. __________________________________________________
3. ____________________________________________________
4. _____________________________________________________

Please tick the boxes of the following barriers as they might apply to you at your workplace:

☐ Other work roles take priority

☐ Lack of time for research

☐ Desire for work/life balance

☐ Lack of funds for research

☐ Lack of skills for research

☐ Lack of suitable backfill

☐ Lack of administrative support

☐ Lack of software for research

☐ Lack of a coordinated approach to research

☐ Other personal commitments

☐ Lack access to equipment for research

☐ Intimidated by research language

☐ Intimidated by fear of getting it wrong

☐ Lack of support from management

☐ Not interested in research

☐ Isolation

☐ Lack of library/internet access

☐ Other (eg, limited exposure to research, lack of access to expertise, statistical analysis, lack of knowledge)

Thinking now of the above barriers you listed, what are the possible solutions for each one?

1. _____________________________________________________
2. _____________________________________________________
3. _____________________________________________________
4. _____________________________________________________

Who will you most likely approach locally to assist you with the above solutions?

1. ______________________________________

2. ______________________________________

What support do you think you will need from the researchers of this study to assist you in performing research/audit?

1. _______________________________________
2. _______________________________________
3. _______________________________________
4. _______________________________________

**F. Personal Motivators:**

What will motivate you to perform research or clinical audit?

1. ______________________________________________
2. ______________________________________________
3. ______________________________________________
4. ______________________________________________

Please tick the boxes of the following motivators as they might apply to you at your workplace:

☐ To develop skills

☐ Increased job satisfaction

☐ Problem identified that needs changing

☐ To keep the brain stimulated

☐ Career advancement

☐ Research encouraged by managers

☐ Links to universities

☐ Mentors available to supervise

☐ Opportunities to participate at own level

☐ Desire to prove a theory or hunch

☐ Dedicated time for research

☐ Colleagues doing research

☐ Grant funds

☐ Forms part of postgraduate study

☐ Research written into role description

☐ Study or research scholarships

☐ Other (eg, to gather evidence that is relevant to practice, to increase knowledge, to keep at the cutting edge, support a new health initiative)

Other, please specify/Or any other comments:

That concludes the questionnaire.

Thank you for your time.

Code:
