# Supplementary material for: Building reproductive health research and audit capacity and activity in the pacific islands (BRRACAP) study: methods, rationale and baseline results
Source: BMC Med Educ. 2014 Jun 19;14:121. doi: 10.1186/1472-6920-14-121 (PMC4069343; doi:10.1186/1472-6920-14-121)

Appendix 2 - Interventions, Tasks and Objective of the BRRACAP Study

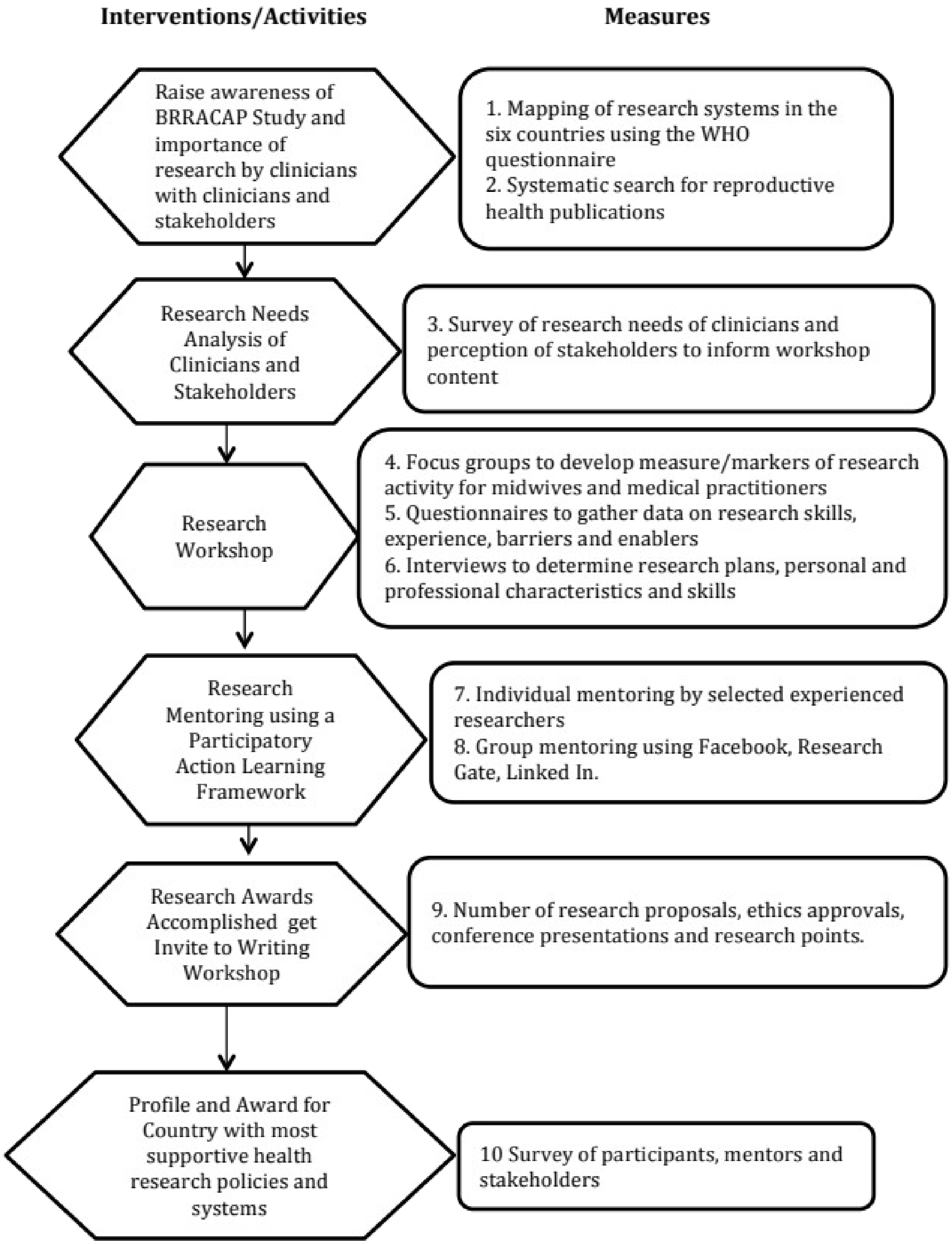

Supplement: Supplementary file 3 [file 1472-6920-14-121-S2.docx]
